# Case Report: A Novel Single Variant TJP2 Mutation in a Case of Benign Recurrent Intrahepatic Cholestasis

**DOI:** 10.1097/PG9.0000000000000087

**Published:** 2021-06-25

**Authors:** Gaël A. Kornitzer, Fernando Alvarez

**Affiliations:** From the * Faculty of Medicine, University of Montreal, Montreal, QC, Canada; † Division of Gastroenterology, Hepatology and Nutrition, CHU Sainte-Justine, Montreal, QC, Canada; ‡ Department of Pediatrics, University of Montreal, Montreal, QC, Canada.

**Keywords:** benign recurrent intrahepatic cholestasis, familial cholestasis, jaundice, liver

## Abstract

Benign recurrent intrahepatic cholestasis (BRIC) is a disease on the spectrum of familial intrahepatic cholestasis caused by homozygous *ABCB11* or *ATP8B1* mutations. In recent years, genetic testing has allowed for discovery of a variety of homozygous or compound heterozygous *TJP2* mutations associated with progressive familial intrahepatic cholestasis and intrahepatic cholestasis of pregnancy. To our knowledge, no cases of BRIC caused by a single variant mutation of *TJP2* have been reported. We describe a 15-year-old female presenting with recurrent episodes of jaundice, vomiting, with intense pruritus, anorexia, and weight loss. Blood work revealed elevated serum conjugated bilirubin and liver enzymes but normal gamma-glutamyl transferase, consistent with BRIC. A genetic panel identified a not previously described single allele mutation in *TJP2* of unknown functional significance. This is the first reported case of a clinical entity resembling BRIC with a heterozygous mutation in *TJP2*, without associated mutations in other cholestasis-related genes.

## INTRODUCTION

Progressive familial intrahepatic cholestasis is a disease causing cholestasis with progressive biliary cirrhosis, caused by autosomal recessive mutations in *ATP8B1*, *ABCB11*, *ABCB4*, and *TJP2* (1,2). *TJP2* is located on chromosome 9q12-q13, and codes for tight junction protein 2 or zonula occludens 2, a scaffold protein involved in epithelial tight junction structure. In the liver, the structure of tight junction impacts bile duct permeability and certain mutations can cause bile acids to leak out and into plasma, resulting in cholestasis (3,4). Carlton et al (3) described a series of 17 patients, 11 with homozygous *TJP2* mutations, with familial hypercholanemia, a rare hepatobiliary disease causing elevated serum bile acids and fat malabsorption. Mutations in *TJP2* have also been associated with cases of remitting or progressive cholestasis. A cohort described by Zhang et al (4) of patients presenting with cholestasis before 1 year had biallelic mutations of *TJP2*. Ge et al (5) reported a case of compound heterozygous *TJP2* mutations in a patient with progressive familial intrahepatic cholestasis initially presenting at 23 months old. Single-allelic mutations in *TJP2* have been potentially linked with cholestasis and episodes of pruritus, usually with precipitation by an acute condition such as drug or toxin ingestion, pregnancy, or infection. In patients with intrahepatic cholestasis of pregnancy, Dixon et al (6) suggested potential heterozygous mutations in *TJP2* in patients with no identifiable mutation in *ABCB4* or *ABCB11*.

Benign recurrent intrahepatic cholestasis (BRIC) is a known clinical entity in the spectrum of familial intrahepatic cholestasis, first described by Summerskill and Walshe (7) in 1959. It is characterized by intermittent episodes of cholestasis with hyperbilirubinemia, accompanied by pruritus, fatigue, anorexia, and steatorrhea, generally presenting in the second decade of life. Inheritance of BRIC is autosomal recessive and 2 types are described in the literature, BRIC1 and BRIC2, associated with mutations in *ATP8B1* and *ABCB11*, respectively (8).

## CASE REPORT

We report a 15-year-old girl who was referred to the Hepatology service at Sainte-Justine’s Hospital for intermittent episodes of jaundice and intense pruritus. Informed consent was obtained from the patient and legal guardians for publication of the following case details. On presentation, the patient had noticeable icterus and complained of nausea, vomiting, and anorexia, and had weight loss of 4.5 kg. Physical exam revealed jaundice and skin excoriations. No hepatosplenomegaly or other stigmata of liver disease were noted. Initial blood work showed elevated serum direct bilirubin of 3.7 mg/dL with total bilirubin of 5.2 mg/dL and alanine aminotransferase of 178 U/L, with normal gamma-glutamyl transferase. Serum bile acids were not measured, as this analysis is not readily available in our center. An abdominal ultrasound performed at this time showed normal liver parenchyma, no biliary duct dilatation, and a normal gallbladder. Three small hyperechoic liver nodules were noted, the largest measuring 5.5 mm and located in segment VIII, resembling benign hemangiomas. On repeat ultrasound and computed tomography scan performed 1 year later, these nodules are not mentioned and liver is noted as normal.

The presenting episode coincided with the start of combined oral contraceptives. Upon discontinuation of the contraceptives, partial improvement of jaundice and pruritus as well as decreasing levels of serum bilirubin were noticed. Later, treatment with rifampin was initiated, leading to total resolution of pruritus. Within 7 months, serum bilirubin had normalized and symptoms completely resolved. Initially, mild elevation of alanine aminotransferase persisted at 60 U/L but on recent blood work is completely normalized. Ursodeoxycholic acid was later started and the patient has had no recurrence of cholestasis in over 10 months. Of note, liver function always remained intact with normal albumin and prothrombin time/international normalized ratio. Liver biopsy and further workup for hepatitis have not been performed due to normalization of bilirubin and aminotransferases, and patient did not have risk factors for nonalcoholic steatohepatitis or other medications that could cause a drug-induced hepatitis.

The patient’s previous medical history was remarkable for neonatal jaundice, as well as 3 to 4 episodes per year of jaundice with pruritus since the age of 4 years, often preceded by minor infections. While we do not have laboratory results from previous episodes of jaundice and thus concomitant Gilbert’s syndrome (*UGT1A1* mutation) cannot be excluded, all blood work performed since referral to our service has shown predominantly conjugated hyperbilirubinemia and, since remission, total bilirubin in addition to direct bilirubin remains completely normalized. In addition, previous episodes were accompanied by intense pruritus, which would not be explained by isolated unconjugated hyperbilirubinemia, suggesting previous episodes were cholestatic in nature. Furthermore, there is no known family history of diagnosed intrahepatic cholestasis; however, similar episodes of remitting and relapsing intense pruritus were reported in second- and third-degree relatives of the father.

A cholestasis-specific genetic panel (PreventionGenetics, Marshfield, WI) was performed, using next-generation and Sanger sequencing technologies, analyzing the following genes: *ABCB11, ABCB4, AKR1D1, ATP8B1, HSD3B7, JAG1, NOTCH2, NR1H4, SERPINA1, SLC25A13, TJP2, VIPAS39,* and *VPS33B*. We identified a single heterozygous variant of unknown significance in *TJP2* (c.3067_3068delinsTT variant), predicted to result in the amino acid substitution p.Ala1023Phe. A single submission for this specific insertion/deletion-type variant exists in the National Institute of Health database of genetic mutations: ClinVar. However, this mutation has not been formally described in the literature and its functional impact remains unknown (9).

The mutation identified in our patient is not located in a domain of the *TJP2* gene whose crystal structure has been studied. As such, we cannot currently hypothesize the functional impact of this mutation on the tight junction protein 2 (9, 10).

## DISCUSSION

The clinical spectrum of intrahepatic cholestasis is a heterogeneous group of diseases in which dysfunction or absence of proteins involved in bile acid homeostasis leads to cholestasis with or without subsequent cirrhosis. Mutations described in these diseases are generally autosomal recessive with varying degrees of penetrance (5, 6, 8). BRIC, specifically, has previously been attributed to recessive mutations in *ATP8B1* and *ABCB11* (8).

The findings in our patient suggest a possible new implication of *TJP2* in BRIC. Further observation of our patient is necessary in order to ascertain the benign, nonprogressive nature of her cholestasis. Should our patient’s condition continue to evolve as benign recurrent cholestasis, as we suspect, this would be the first reported case of a clinical entity resembling BRIC potentially associated with an autosomal dominant *TJP2* mutation. These findings further reinforce the concept that progressive familial intrahepatic cholestasis, BRIC, and intrahepatic cholestasis of pregnancy are diseases that belong to a single spectrum and that phenotype may vary according to zygosity as well as type and amount functional change associated with different mutations.

## ACKNOWLEDGMENTS

G.A.K. conducted the literature review, completed chart review for the patient presented, as well as drafted and finalized the article for this case report. F.A. is the treating physician of the patient presented in this case report. He completed chart review, supervised redaction of the article and approved the article to be submitted.
